# Factors associated with prolonged length of stay for elective hepatobiliary and neurosurgery patients: a retrospective medical record review

**DOI:** 10.1186/s12913-017-2817-8

**Published:** 2018-01-05

**Authors:** Siu Yin Lee, Soo-Hoon Lee, Jenny H. H. Tan, Howard S. L. Foo, Phillip H. Phan, Alfred W. C. Kow, Sein Lwin, Penelope M. Y. Seah, Siti Zubaidah Mordiffi

**Affiliations:** 1 National University Hospital, 5 Lower Kent Ridge Road, Singapore, 119074 Singapore; 2 Old Dominion University, 1 Old Dominion University, Norfolk, VA 23529 USA; 3 Ngee Ann Polytechnic, 535 Clementi Road, Singapore, 599489 Singapore; 4 Woodlands Health, 9 Maxwell Road, Singapore, 069112 Singapore; 5 Johns Hopkins University, 100 International Drive, Baltimore, MD 21202 USA

**Keywords:** Prolonged length of stay, Elective surgery, Preoperative, Intraoperative, Postoperative care, Ambulatory services

## Abstract

**Background:**

Patients with prolonged length of hospital stay (LOS) not only increase their risks of nosocomial infections but also deny other patients access to inpatient care. Hepatobiliary (HPB) malignancies have some of highest incidences in East and Southeast Asia and the management of patients undergoing HPB surgeries have yet to be standardized. With improved neurosurgery techniques for intracranial aneurysms and tumors, neurosurgeries (NS) can be expected to increase. Elective surgeries account for far more operations than emergencies surgeries. Thus, with potentially increased numbers of elective HPB and NS, this study seeks to explore perioperative factors associated with prolonged LOS for these patients to improve safety and quality of practice.

**Methods:**

A retrospective cross-sectional medical record review study from January 2014 to January 2015 was conducted at a 1250-bed tertiary academic hospital in Singapore. All elective HPB and NS patients over 18 years old were included in the study except day and emergency surgeries, resulting in 150 and 166 patients respectively. Prolonged LOS was defined as above median LOS based on the complexity of the surgical procedure. The predictor variables were preoperative, intraoperative, and postoperative factors. Student’s t-test and stepwise logistic regression analyses were conducted to determine which factors were associated with prolonged LOS.

**Results:**

Factors associated with prolonged LOS for the HPB sample were age and admission after 5 pm but for the NS sample, they were functional status, referral to occupational therapy, and the number of hospital-acquired infections.

**Conclusion:**

Our findings indicate that preoperative factors had the greatest association with prolonged LOS for HPB and NS elective surgeries even after adjusting for surgical complexity, suggesting that patient safety and quality of care may be improved with better pre-surgery patient preparation and admission practices.

**Electronic supplementary material:**

The online version of this article (10.1186/s12913-017-2817-8) contains supplementary material, which is available to authorized users.

## Background

Length of stay (LOS) is an important measure of resource utilization as patients with prolonged LOS disproportionately account for the consumption of more hospital resources [1]. Prolonged LOS, which is defined as inpatient stay that exceeds the expected LOS for a certain procedure [2], unnecessarily utilizes hospital beds, contributing to capacity shortage. Inpatient bed shortage delays elective admissions and increases emergency department boarding, which denies critically ill patients timely access to treatment, as inpatient care conventionally begins only after the patient arrives at the assigned inpatient ward [3, 4]. Patients with prolonged LOS are also at higher risks of nosocomial infections and unplanned readmissions [5, 6]. In short, prolonged LOS disadvantages both hospitals and patients [7].

Surgical services represent approximately 30% of all hospital expenses [8] and elective surgeries are far more common than emergency operations [9]. Unlike emergency admissions where it is difficult for hospital administrators to influence pre-admission factors, knowing the factors that increase LOS in elective surgeries can allow hospital staff to proactively manage the delivery of care and demand for beds [10]. While past studies have explored the factors associated with prolonged LOS after cardiac [11], laparoscopic cholecystectomy [12–14], and spine [15, 16] surgeries, no studies have explored the factors that lead to prolonged LOS in hepatobiliary (HPB) and neurosurgeries (NS). HPB malignancies, liver tumors, and other liver diseases have the highest incidences in Asia and Southeast Asia compared to the rest of the world due to widespread hepatitis B virus infections [17]. Surgery remains the only option for treatment. However, HPB procedures are variable and their care management practices are not standardized [18]. Consequently, the LOS for HPB surgical patients varies widely. For NS, the demand for such surgeries is expected to increase because of improved surgical procedures in craniotomy and cranioplasty for intracranial aneurysms, brain tumors, and other cerebrovascular diseases as well as aging populations and changes in lifestyle in developed countries, including Asia [19]. By explicating factors associated with prolonged LOS for HPB and NS elective surgeries, hospitals can design better patient management services [20]. In sum, studying these two types of high demand elective surgical procedures is more likely to lead to meaningful reductions in the overuse of bed capacity.

Multiple factors at the patient and systems level have been associated with LOS in a variety of disease settings. For example, common preoperative patient factors associated with prolonged LOS are the patient’s demographics and presenting conditions at the time of admission [21]. Studies have shown that age, gender, functional status, diagnostic or illness factors, and the major procedure group for surgical patients influenced LOS [2, 3, 12–14, 22]. Specifically, age was related to prolonged LOS for patients undergoing aortic surgery [23]. In general, elderly patients undergoing a major surgery face higher risks of bleeding and other physiological dysfunctions that may slow recovery and contribute to prolonged LOS [24]. Seicean et al. [15] found that elective spine surgery patients with higher body-mass index (BMI) reported poorer outcomes. The Braden score on admission, which identifies patients at higher risk for pressure ulcers, was found to predict prolonged LOS for patients undergoing liver transplantation [25], while malnutrition on admission slows recovery [26]. Patients who are frail and cannot ambulate independently have a low functional status score and were reported to have prolonged LOS following elective lumbar spine and arterial vascular surgeries [27, 28]. Comorbidity also predicted prolonged LOS [29]. Gruskay et al. [16] found that the total number of comorbidities predicted prolonged LOS after elective lumbar surgery. Likewise, the number of abnormal laboratory results (labs) indicate the severity of patients’ health status, which influences how fast they will recover after surgery. Fall risk is measured by assessing the patient’s level of confusion, dizziness, altered elimination, and difficulty with mobility at admission [30, 31]. Preoperative fall risks have been found to predict postoperative falls, functional decline, and surgical complications, all of which prolonged LOS [32]. Occupational therapy (OT) improves walking capacity, muscle strength, flexibility, balance, and endurance so patients can quickly return to active lifestyles [33, 34]. However, patients need a minimum capacity to do OT, and preoperative physiotherapy (PT) has been shown to improve exercise tolerance for patients undergoing lung transplantation [35].

Preoperative hospital administrative system factors that contribute to LOS include delays in investigation and procedures [36] as well as the day and time of admission [37]. For example, patients admitted during weekends, on the eve of public holidays, or after 5 pm often face delays in the initial physical examination and diagnostic tests due to diminished availability of staff and expertise. In general, perioperative patient care after office hours is limited by a reduced clinical crew. Consequently, the day and time of admission have been associated with increased LOS, preventable adverse events, and mortality of patients with pulmonary embolism and myocardial infarction [37, 38].

Intraoperative variables that affect LOS include the duration of preoperative preparation [39] and operation time [2]. For example, for patients undergoing surgery for endocrine tumor, the duration of preoperative preparation for anesthesiologic management, fluid management, medications involving anti-hypertensive medications, alpha, beta, or calcium channel blockers, and antibiotic prophylaxes, were associated with prolonged LOS [39]. Delays or conflicts in preoperative preparation may lead to a cancellation of the surgery, which further increases LOS. Chang et al. [23] found that operations lasting more than 5 h, which may be related to intraoperative blood loss, were associated with prolonged LOS for patients undergoing aortic surgery.

Postoperative variables that affected LOS include the number of complications after surgery and hospital-acquired infections (HAI) [2, 23, 40, 41]. For example, complications such as urinary tract infection and pneumonia led to prolonged LOS following cervical discectomy and fusion [41]. Postoperative treatment for wound or surgical site infections [23], polypharmacy [40, 42] where multiple drug consumption increased the risk of drug interactions and adverse reactions, as well as use of dialysis, ventilator support, and catheter insertions [3] also contributed to LOS. Other hospital acquired nosocomial infections include infections from clostridium difficile colitis [43] or methicillin-resistant *Staphylococcus aureus* [6]. Research has also shown that discharge planning affects LOS, where discharge to a nursing home was associated with prolonged LOS by 89% for surgical patients compared to being discharged to home [3, 36].

In sum, there is broad literature to suggest that multifactor perioperative pathways, excluding the operation procedure, have significant impact on prolonged LOS. However, current studies exploring such factors have only focused on partial perioperative pathways. For example, Freitas et al. [7], Reponen et al. [44], and Lukasiewicz et al. [21] focused on the patients’ preoperative clinical conditions, Morimoto et al. [13] investigated preoperative and intraoperative clinical factors, while Collins et al. [2] considered intraoperative and postoperative factors. These studies could not rule out confounding factors from the partial pathway that could otherwise be non-significantly related to prolonged LOS if the entire perioperative pathway was considered. In this study, we examined perioperative factors in the entire surgical pathway. By assessing the simultaneous impact of these factors on prolonged LOS, hospitals can set reasonable preoperative expectations for patients and the healthcare team, establish efficient operative protocols, or plan postoperative treatment care and improve discharge planning for patients. Our approach is more likely to yield a comprehensive surgical services protocol for the entire perioperative care continuum for elective HPB and NS patients.

The objective of this study is to assess the association between preoperative, intraoperative, and postoperative factors on prolonged LOS after adjusting for surgical complexity for a sample of elective HPB and NS patients. Since surgeries are more susceptible to the risk of adverse events than non-surgical procedures [45, 46] and elective surgeries outnumber emergency procedures, we contribute to the literature on surgical safety and quality of care with greater impact for potential policy interventions that influence LOS. In the next section, we describe the setting and design of the study, followed by a description of the patient population, the data collection process, the variables, and the statistical analyses. We then report and discuss the results. We conclude with a discussion on the applications of the findings.

## Methods

### Design and setting

We conducted a retrospective cross-sectional medical record review of elective HPB and NS patients admitted between January 2014 and January 2015 to a 1250-bed tertiary academic center in Singapore, which was the first to receive the Joint Commission International accreditation in the country (http://www.nuhs.edu.sg/about-us/corporate-profile.html). Our study site mirrors the challenge of bed shortages in public hospitals in urban environments as well as incidences of HPB malignancies and demand for NS due to an aging population [47].

### Participants

All patients over 18 years old who were admitted between January 2014 and January 2015 for elective HPB and NS procedures via the hospital’s specialist outpatient clinics were included in the study. Patients who were admitted for emergency surgeries and day surgeries, such as repairs of inguinal hernia, anal fissure, appendectomy, and cholecystectomy, were excluded from the study. The samples for this study were 150 HPB and 166 NS patients.

### Data collection

One nurse administrator and one operations administrator, who were not involved in surgeries, extracted preoperative, intraoperative, and postoperative information from the medical records with the assistance of five registered nurses. This study was IRB approved by the National Healthcare Group Domain-Specific Review Board (DSRB 2015/00229).

### Measures

The outcome variable in this study was prolonged LOS. The Singapore Ministry of Health provides a Table of Surgical Procedures (TSP), equivalent to the American Society of Anesthesiologists (ASA) class codes, which is an exhaustive list of surgical procedures ranked by complexity in ascending order from 1A to 7C for insurance claims [48]. We defined prolonged LOS as the days of hospital stay above the median LOS of the TSP, as defined by Collins et al. [2] and Morimoto et al. [13], as shown in Additional file 1. The complexity of surgery influences prolonged LOS and so shortened LOS is defined as LOS below the median LOS of the TSP.

Coding of the predictor variables is provided in Additional file 2. Since prolonged LOS for HPB and NS have not been previously studied, we looked to the literature on other types of surgeries to determine the appropriate predictor variables on prolonged LOS. Preoperative factors found in other types of surgeries, as discussed earlier, included: (1) patients’ demographics (age, gender, and race), (2) patients’ presenting conditions at admission (BMI, Braden Scale [49], Nutrition Score [50], functional status measured by the Katz Index [51], smoking history, number of comorbidities, number of abnormal labs, and number of fall risks), and (3) hospital administrative system factors (weekend admission, admission after 5 pm, referrals to PT and OT, and number of delays in diagnostic tests and appointments).

Intraoperative factors found in other types surgeries that influenced LOS, as discussed earlier, included surgical factors related to (1) preparation duration, which is the time interval between the patient’s entry into the operating theatre and incision based on the TSP, (2) operation duration, which is the time interval between incision to closure based on the TSP, and (3) operation ending after 5 pm.

Finally, postoperative factors found in other types surgeries that influenced LOS, as discussed earlier, included: (1) post-surgery inpatient factors (number of insertions, number of HAI, and number of medications) and (2) discharge planning (discharged after 5 pm).

### Statistical analysis

As our study included many predictor variables with small sample sizes, we analyzed the data in two steps. First, we conducted Student’s t- and χ^2^-tests on the continuous and dichotomous variables respectively, to identify factors that statistically differentiated between prolonged and shortened LOS. Next, we used these factors in a stepwise logistic regression analysis to derive the predictive model of prolonged LOS. We conducted a backward logistic regression analysis to verify the results. The statistical analyses were performed using SPSS version 23 [52] and statistical significance was assumed at *p* < 0.05 throughout this study.

## Results

The data reports that the median LOS for both the HPB and NS samples was 6 days. The interquartile range (IQR), which indicates the 25th and 75th percentile values, for the HPB sample was 2–8 days while the IQR for the NS sample was 4–9 days. The mean (± standard deviation) LOS for the HPB and NS samples were 7.18 (± 8.01) and 10.99 (± 21.02) days respectively.

Table 1 reports the factors that significantly differentiated between prolonged and shortened LOS for the HPB sample. Among the preoperative factors, they were age and admission after 5 pm; among the intraoperative factors, they were preparation duration, operation duration, and operations ending after 5 pm; and among the postoperative factors, they were the number of insertions and the number of HAI.

**Table 1:** Student’s t- or χ^2^-tests of differences between below and above median LOS for predictors in the HPB Sample

Means or Percentages	At Below LOS Median of the TSP	At Above LOS Median of the TSP	*p*-value
Preoperative Factors:			
Age ± s.d.	55.54 ± 14.29	64.62 ± 10.97	< 0.001
Gender: Male (n)	53.00% (35)	65.20% (43)	0.11
Race: Chinese (n)	60.60% (40)	57.60% (38)	0.43
Unhealthy BMI (n)	67.40% (29)	65.20% (15)	0.53
Braden Scale ± s.d.	22.73 ± 0.93	21.96 ± 2.12	0.06
Nutrition Score ± s.d.	0.36 ± 0.91	0.73 ± 1.40	0.18
Functional Status ± s.d.	0.26 ± 0.91	0.88 ± 1.73	0.06
Smoking History (n)	15.60% (7)	17.90% (5)	0.52
Number of Comorbidities ± s.d.	1.39 ± 1.10	1.75 ± 1.21	0.18
Number of Abnormal Labs ± s.d.	2.49 ± 1.68	3.23 ± 1.58	0.08
Number of Fall Risks ± s.d.	0.05 ± 0.21	0.15 ± 0.36	0.13
Weekend Admissions (n)	9.10% (6)	9.10% (6)	0.99
Admissions After 5 pm (n)	18.20% (12)	50.00% (33)	< 0.001
Referral to OT (n)	9.10% (4)	14.80% (4)	0.36
Referral to PT (n)	18.20% (8)	29.60% (8)	0.20
Number of Delays ± s.d.	1.11 ± 1.29	2.19 ± 3.76	0.66
Intraoperative Factors:			
Preparation Duration ± s.d.	1:02:51 ± 0:26:16	1:14:35 ± 0:29:44	0.02
Operation Duration ± s.d.	2:53:09 ± 2:09:03	3:48:42 ± 2:36:39	0.03
Operation Ends After 5 pm (n)	19.70% (13)	40.90% (27)	0.01
Postoperative Factors:			
Number of Insertions ± s.d.	1.89 ± 2.16	1.18 ± 1.77	0.02
Number of HAI ± s.d.	0.20 ± 1.21	0.48 ± 0.85	0.01
Number of Medications ± s.d.	0.18 ± 0.45	0.44 ± 0.85	0.46
Weekend Discharge (n)	37.90% (25)	28.80% (19)	0.18
Discharged After 5 pm (n)	12.10% (8)	19.70% (13)	0.17

*LOS* length of stay

*HPB* hepatobiliary

*TSP* table of surgical procedure

*s.d* standard deviation

*BMI* body-mass index

*Unhealthy BMI* BMI outside the 18.5–23 range

*OT* occupational therapy

*PT* physiotherapy

*HAI* hospital-acquired infections

Table 2 reports the factors that significantly differentiated between prolonged and shortened LOS for the NS sample. Among the preoperative factors, they were age, gender, functional status, the number of abnormal labs, the number of fall risks, admission after 5 pm, and referrals to PT and OT. Among the intraoperative factors, it was operation duration; and among the postoperative factors, they were the number of HAI, the number of medications, and discharged after 5 pm.

**Table 2:** Student’s t- or χ^2^-tests of differences between below and above median LOS for predictors in the NS sample

Means or Percentages	At Below LOS Median of the TSP	At Above LOS Median of the TSP	p-value
Preoperative Factors:			
Age ± s.d.	46.56 ± 17.38	52.87 ± 15.91	0.03
Gender: Male (n)	31.80% (21)	47.40% (37)	0.04
Race: Chinese (n)	62.10% (41)	67.90% (53)	0.29
Unhealthy BMI (n)	78.80% (26)	64.50% (20)	0.16
Braden Scale ± s.d.	22.42 ± 1.23	21.39 ± 2.79	0.06
Nutrition Score ± s.d.	0.16 ± 0.50	0.38 ± 0.92	0.42
Functional Status ± s.d.	0.14 ± 0.68	1.27 ± 1.91	< 0.001
Smoking History (n)	10.50% (4)	12.50% (5)	0.53
Number of Comorbidities ± s.d.	0.95 ± 0.93	1.24 ± 1.02	0.19
Number of Abnormal Labs ± s.d.	1.65 ± 1.10	2.75 ± 1.35	< 0.001
Number of Fall Risks ± s.d.	0.32 ± 0.85	0.57 ± 0.70	0.02
Weekend Admissions (n)	34.80% (23)	33.30% (26)	0.49
Admissions After 5 pm (n)	33.30% (22)	54.70% (41)	0.01
Referral to OT (n)	11.10% (4)	32.40% (11)	0.03
Referral to PT (n)	13.90% (5)	35.30% (12)	0.04
Number of Delays ± s.d.	1.40 ± 1.73	1.39 ± 1.69	0.60
Intraoperative Factors:			
Preparation Duration ± s.d.	1:07:43 ± 0:24:03	1:15:08 ± 0:26:48	0.09
Operation Duration ± s.d.	1:40:52 ± 0:58:15	2:27:16 ± 1:55:03	<0.001
Operation End After 5 pm (n)	15.20% (10)	17.90% (14)	0.41
Postoperative Factors:			
Number of Insertions ± s.d.	0.85 ± 1.04	1.18 ± 1.35	0.24
Number of HAI ± s.d.	0. 05 ± 0.23	0.55 ± 0.83	< 0.001
Number of Medications ± s.d.	0.08 ± 0.36	0.56 ± 0.82	< 0.001
Weekend Discharge (n)	37.90% (25)	24.40% (19)	0.06
Discharged After 5 pm (n)	18.20% (12)	36.80% (28)	0.01

*LOS* length of stay

*NS* neurosurgery

*TSP* table of surgical procedure

*s.d* standard deviation

*BMI* body-mass index

*Unhealthy BMI* BMI outside the 18.5–23 range

*OT* occupational therapy

*PT* physiotherapy

*HAI* hospital-acquired infections

In the logistic regression models, Table 3 reports that only preoperative factors (age and admission after 5 pm) predicted prolonged LOS in the HPB sample after adjusting for surgical complexity. Table 4 reports that preoperative factors (functional status and referrals to OT) predicted prolonged LOS in the NS sample after adjusting for surgical complexity. The only postoperative factor to predict prolonged LOS in the NS sample was the number of HAI.

**Table 3:** Stepwise logistic regression on prolonged LOS for HPB sample

Predictors^b^	Exp(B)	p-value
Constant	0.01	0.002
Preoperative Factors:		
Age	1.06	0.01
Admissions After 5pm^a^	4.35	0.01

*LOS* length of stay

*HPB* hepatobiliary surgery

^a^Reference group

^b^Statistically non-significant factors omitted from the table

**Table 4:** Stepwise logistic regression on prolonged LOS for NS sample

Predictors^b^	Exp(B)	p-value
Constant	0.16	< 0.001
Preoperative Factors:		
Functional Status	1.93	0.02
Referral to OT^a^	7.10	0.01
Postoperative Factors:		
Number of HAI	19.89	0.01

*LOS* length of stay

*NS* neurosurgery

*OT* occupational therapy

*HAI* hospital-acquired infections

^a^Reference group

^b^Statistically non-significant predictors are omitted from the table

## Discussion

Among the preoperative, intraoperative, and postoperative factors that statistically differentiated between prolonged and shortened LOS in our samples, the logistic regression model reports that preoperative factors in the surgical pathway related to patient factors were the most predictive of prolonged LOS. Specifically, age was associated with prolonged LOS for the HPB sample, while functional status and referrals to OT were associated with prolonged LOS for the NS sample. In line with Chang’s [23] study on aortic surgery, age was related to prolonged LOS. Elderly patients face physiological dysfunctions from major surgeries that may slow recovery and delay hospital discharge [24]. Age is also a multifactorial demographic characteristic that is related to functional status [27], comorbidities [24, 42], polypharmacy [42], and postoperative complications [23].

For the NS sample, patient factors predictive of prolonged LOS from the logistic regression were functional status and referrals to OT. Similar to Partridge’s [28] study on arterial vascular surgery, our data showed that low functional status and frail patients who needed OT had prolonged LOS. Our results may suggest that physiologically vulnerable patients may need additional care plans to improve their walking capacity, muscle strength, flexibility, balance, or endurance, thus prolonging LOS.

The implications of knowing that age, functional status, and frailty needing OT may be associated with prolonged LOS for HPB and NS patients are that hospitals could implement evidence-based policies to prepare such patients for surgery to increase patient safety and reduce LOS. For example, hospitals could consider adopting preoperative protocols such as home visits and enhanced recovery after surgery (ERAS) protocols [53] to prepare elderly or frail patients for upcoming elective surgeries [54]. By making patients more aware of their recovery pathways, such protocols empower patients and their families to take responsibility (e.g., for early and frequent ambulation) for their own well-being.

The logistic regression model for the HPB sample indicates that among systems factors, admission after 5 pm was associated with prolonged LOS, in line with Earnest’s [37] study. Admission to the hospital for surgery after 5 pm is an indicator of an operational inefficiency since no elective surgeries occurred at night at this academic center. Admission after 5 pm not only added another day of hospital stay but patients also face delays in diagnostic tests as well as limited inpatient care due to the diminished availability of staff and expertise at night. This finding may suggest that hospitals could consider establishing a policy of not admitting patients for elective surgeries after 5 pm to avoid an additional day at the hospital and preventing delays. This is particularly important for the first surgery of the day since delays will cause accumulating scheduling cascades through the day.

The logistic regression model showed that the number of HAI, a postoperative factor, predicted prolonged LOS for the NS sample. This result suggests the importance of reducing surgical site infections and other complications after surgery. Specifically, post-surgical care should focus on wound support and better monitoring by the team for pneumonia and nosocomial infections. Perhaps, hospitals could consider postoperative procedures such as Team Strategies and Tools to Enhance Performance and Patient Safety (TeamSTEPPS) to improve teamwork in patient monitoring among the inpatient care team. Evidence suggests that such protocols can reduce HAI to improve patient safety [55].

A limitation of this study is that the data was hand collected from case notes, which increases the risk of translation error. Random quality checks mitigated this risk. The resource intensity of case notes review meant that we could only collect one complete year of data, which limits the predictive power of our results. A second limitation is the cross-sectional design, which restricts a determination of a causal relationship among the variables. This limitation presents a future research opportunity with longitudinal studies. Finally, our results came from a single site even though the findings are broadly consistent with previous studies in other settings, as noted in the earlier discussion.

## Conclusion

Our findings indicate that preoperative patient factors related to age, functional status, and frailty requiring OT as well as system factors related to admission after 5 pm prolonged LOS for patients in our HPB and NS samples. Postoperative factor related to the number of HAI was associated with prolonged LOS for the NS sample. The results suggest that patient safety and quality of care may be improved with better pre-surgery patient preparation and admission practices as well as improved patient monitoring after surgery through better teamwork to reduce HAI. Our results may offer insights for hospital administrators and clinicians to be conscious of patient and system level factors that could lead to prolonged LOS in elective HPB and NS surgeries since no studies have explored perioperative factors for the entire surgical pathway in these two types of high demand elective surgeries. We hope that our findings help hospitals design better patient management services by setting reasonable preoperative expectations for patients and the healthcare team, establishing efficient admission protocols, and providing effective postoperative treatment care that improve meaningful reductions in LOS.

## Additional files

Additional file 1: Median LOS in days according to surgical complexity. (DOCX 19 kb)

Additional file 2: Coding of the predictor variables. (DOCX 28 kb)

## Abbreviations

ASA: American Society of Anesthesiologists
BMI: Body-mass index
ERAS: Enhanced recovery after surgery
HAI: Hospital-acquired infections
HPB: Hepatobiliary
IQR: Interquartile range
LOS: Length of stay
NS: Neurosurgery
OT: Occupational therapy
PT: Physiotherapy
TeamSTEPPS: Team Strategies and Tools to Enhance Performance and Patient Safety
TSP: Table of surgical procedure
