# Supplementary material for: Factors associated with prolonged length of stay for elective hepatobiliary and neurosurgery patients: a retrospective medical record review
Source: BMC Health Serv Res. 2018 Jan 5;18:5. doi: 10.1186/s12913-017-2817-8 (PMC5755148; doi:10.1186/s12913-017-2817-8)
Supplement: Supplementary file 1 — Median LOS in days according to surgical complexity. (DOCX 19 kb) [file 12913_2017_2817_MOESM1_ESM.docx]

Additional file 1: Median LOS in Days According to Surgical Complexity

|  | **Median LOS in days (IQR, n)** | |
| --- | --- | --- |
| **Table of Surgical Procedure (TSP)** | **HPB Sample (n = 150)** | **NS Sample (n = 166)** |
| TSP ≤ 4 | 2 (IQR = 1-5, n = 73) | 4 (IQR = 1-7.25, n=18) |
| TSP = 5 | 7 (IQR = 5-8, n = 31) | 3.5 (IQR = 1-7, n = 34) |
| TSP = 6 | 7 (IQR = 4.5-9.5, n = 25) | 5 (IQR = 4-8, n = 67) |
| TSP = 7 | 9 (IQR = 7-14, n = 31) | 8 (IQR = 7-14, n = 34) |

LOS = length of stay

IQR = interquartile range between 25^th^ percentile and 75^th^ percentile

HPB = hepatobiliary

NS = neurosurgery
