# Supplementary material for: Factors associated with prolonged length of stay for elective hepatobiliary and neurosurgery patients: a retrospective medical record review
Source: BMC Health Serv Res. 2018 Jan 5;18:5. doi: 10.1186/s12913-017-2817-8 (PMC5755148; doi:10.1186/s12913-017-2817-8)
Supplement: Supplementary file 2 — Coding of the predictor variables. (DOCX 28 kb) [file 12913_2017_2817_MOESM2_ESM.docx]

Additional file 2: Coding of the Predictor Variables

| **Continuous Variables:** | |
| --- | --- |
| Age | |
| Braden Scale (ranges from 6-23 where a lower score indicates a higher level of risk of developing pressure ulcers and a score of 19 or higher indicates low risk requiring no treatment) | |
| Nutrition Score was measured using a Global Assessment tool that evaluates each patient through a physical examination on weight and dietary intake changes, gastrointestinal symptoms, and functional capacity for evidence of fat depletion, muscle wasting and nutrition-related edema. | |
| Functional Status is measured by the Katz Index, which is the total number of the following functional states in which the patient requires help at admission:  (a) bathing  (b) dressing  (c) toileting  (d) transfer, and  (e) feeding | |
| Number of Comorbidities is the total number of following presenting conditions in which the patient has at admission:  (a) anemia  (b) hypertension  (c) liver cirrhosis  (d) diabetes mellitus  (e) chronic obstructive pulmonary disease (COPD)  (f) infection, and  (g) other comorbidities | |
| Number of Abnormal Labs is the total number of the following lab results in which the patient obtained at admission that fell outside the World Health Organization’s definition for normal range:  (a) urea  (b) creatinine  (c) potassium  (d) hemoglobin  (e) hematocrit  (f) serum albumin  (g) leucocytes/total white-blood count (TWC)  (h) prothrombin time  (i) International Normalized Ratio (INR)  (j) other abnormal laboratory results | |
| Number of Fall Risks is total number of following presenting states in which the patient exhibited at admission in confusion and difficulty with mobility from having:  (a) altered mental states  (b) dizziness  (c) altered elimination, and  (d) lower limb weakness | |
| Number of Delays is the total number of the following diagnostic tests or appointments in which the patient faced delays at admission:   1. computerized tomography (CT) scan 2. magnetic resonance imaging (MRI) 3. X-ray 4. other equipment 5. medical social worker (MSW) 6. physiotherapist (PT) 7. occupational therapist (OT)/speech therapist 8. dietitian 9. other allied healthcare workers | |
| Number of Insertions is the total number the following insertions administered to the patient during the hospital stay:  (a) indwelling catheter (IDC)  (b) intravenous catheter (IVC)  (c) surgical drain  (d) nasogastric tube (NGT)  (e) oxygen (O_2_)  (f) central venous pressure (CVP)  (g) intra-arterial line (IA)  (h) calf pump | |
| Number of hospital-acquired infections (HAI) is the total number of the following HAI experienced by the patient during the hospital stay:   1. Falls 2. Pressure ulcer 3. Urinary Tract Infections (UTI) 4. Phlebitis 5. Pneumonia 6. Wound/Surgical Site infection 7. Surgical complications 8. Other HAI | |
| Number of Medications is the total number of the following medications administered to the patient during the hospital stay:   1. Diuretics 2. Warfarin 3. Intravenous (IV) antibiotics 4. Antibiotics 5. Anti-platelets 6. Anti-epileptics 7. Angiotensin-converting enzyme (ACE) inhibitors 8. Other Meds | |
| **Categorical Variables:** | |
| Gender | 1=’male’  0=’female’ |
| Race | 1=’Chinese’  0=’other races’ |
| Unhealthy BMI was coded in accordance to the Singapore population profile (Health Promotion Board, 2016) | 0=’healthy BMI between 18.5-23’  1=’other BMI values’ |
| Smoking History | 1=’ever smoked’  0=’never smoked’ |
| (1) Weekend Admission  (2) Admissions After 5pm  (3) Referral to occupational therapist (OT)  (4) Referral to physiotherapist (PT)  (5) Operation ends after 5pm  (6) Weekend Discharge  (7) Discharged after 5pm | 1=’yes’  0=’no’ |
